# Suicide Statistics

**Published:** 1882-06

**Authors:** 


### SUICIDE STATISTICS.

The *Insurance Chronicle* has made up from newspaper reports a series of tables of the suicides in this country during the year ending March 31st last, which are quite curious and interesting withal. The numb is 817. Supposing that this includes only half the actual suicides—an old Philadelphia undertaker always carried a crooked needle with which to sew up throats of "things" in a quiet way—there are 32 for every million inhabitants, which is probably near the truth.

Of the 817 there were 643 males and 174 females, 349 Americans, 277 Germans, 77 Irish, 13 Scotch, 234 husbands, 98 wives, 144 bachelors, 56 maids, 28 widows and 7 divorced persons. As to the time, 300 passed on in spring, 232 in fall, 186 in summer, and 93 in winter. This shows that "steady weather" is bad for business. Even temperature seems to be bad for it all around, for there are comparatively few cases reported in the South, Ohio had 133, the most of any State, and then followed 96 in Illinois, 57 in New York, 55 in Indiana, 51 in Michigan, and so on.

New England had 33, of which number 8 were in Massachusetts and 10 in Maine. Eight of the Southern States had but 35. As to methods, 223 shot off their mortal coils, 181 poisoned themselves, 158 hung, 94 drowned, 74 chopped their throats, 21 jumped from or in front of railroad trains, and one poor chap scalded himself. Shooting was the favorite with men by great odds, but women, who like to have their cadavers well ordered, favored poison; 58 tried the cup, while 33 took suspension, 27 drowned, and only 15 shot.

The occupations of 387 of the number were given, and of these 104 were farmers, 41 merchants, 24 mechanics, and 22 laborers. This goes to show that out-door work does not prevent despondency. Among causes, family troubles is first, with 88, and then come insanity 77, dissipation 77, sickness 76, love 51, and money troubles 45. The ages run all the way from 11 to 100, and a curious thing appears in a largely increased number at the "corners."

Beginning with 25, every fifth year of age after, up to 60, was marked by a large number of cases, and, in fact, between the two ages mentioned, more people sent themselves home in three years than in all the others. The greatest number of one age was 21 at 40. There were 90 cases between 20 and 30 years of age, 60 between 30 and 40, between 40 and 50 there were 56, between 50 and 60 there were 49, and under 20 there were 40.

